# Interactions Between Variation in Candidate Genes and Environmental Factors in the Etiology of Schizophrenia and Bipolar Disorder: a Systematic Review

**DOI:** 10.1007/s12035-017-0708-y

**Published:** 2017-08-18

**Authors:** Błażej Misiak, Filip Stramecki, Łukasz Gawęda, Katarzyna Prochwicz, Maria M. Sąsiadek, Ahmed A. Moustafa, Dorota Frydecka

**Affiliations:** 1 Department of Genetics, Wroclaw Medical University, 1 Marcinkowski Street, 50-368 Wroclaw, Poland; 2 Department of Psychiatry, Wroclaw Medical University, 10 Pasteur Street, 50-367 Wroclaw, Poland; 3 Department of Psychiatry and Psychotherapy, University Medical Center Hamburg-Eppendorf, Hamburg, Germany; 4 II Department of Psychiatry, Medical University of Warsaw, Warsaw, Poland; 5 Institute of Psychology, Jagiellonian University, Krakow, Poland; 6 School of Social Sciences and Psychology, Marcs Institute of Brain and Behaviour, Western Sydney University, Penrith, NSW Australia

**Keywords:** Psychosis, Bipolarity, Gene polymorphism, Gene × environment interaction

## Abstract

Schizophrenia and bipolar disorder (BD) are complex and multidimensional disorders with high heritability rates. The contribution of genetic factors to the etiology of these disorders is increasingly being recognized as the action of multiple risk variants with small effect sizes, which might explain only a minor part of susceptibility. On the other site, numerous environmental factors have been found to play an important role in their causality. Therefore, in recent years, several studies focused on gene × environment interactions that are believed to bridge the gap between genetic underpinnings and environmental insults. In this article, we performed a systematic review of studies investigating gene × environment interactions in BD and schizophrenia spectrum phenotypes. In the majority of studies from this field, interacting effects of variation in genes encoding catechol-*O*-methyltransferase (*COMT*), brain-derived neurotrophic factor (*BDNF*), and FK506-binding protein 5 (*FKBP5*) have been explored. Almost consistently, these studies revealed that polymorphisms in *COMT*, *BDNF*, and *FKBP5* genes might interact with early life stress and cannabis abuse or dependence, influencing various outcomes of schizophrenia spectrum disorders and BD. Other interactions still require further replication in larger clinical and non-clinical samples. In addition, future studies should address the direction of causality and potential mechanisms of the relationship between gene × environment interactions and various categories of outcomes in schizophrenia and BD.

## Introduction

Schizophrenia and bipolar disorder (BD) represent complex and multidimensional phenotypes with high heritability rates, exceeding 80% in twin studies [1–3]. However, the concept of schizophrenia and BD as monogenic disorders was abandoned many years ago, since genome-wide association studies (GWASs) have revealed that the risk of schizophrenia and BD is conferred by a large number of alleles with small effect sizes, all of them explaining only some part of susceptibility [4, 5]. In addition, GWASs have not confirmed several findings from studies based on a candidate gene approach [4, 5]. The polygenic risk score that was developed based on GWASs has been found to mediate nearly 20% of familial liability for schizophrenia, suggesting an important role of non-genetic factors [6]. It has been also reported that some cases of schizophrenia might be attributed to rare structural aberrations that are characterized by moderate or large effects and include copy number variations, deletions, duplications, and translocations [7]. To make things more complex, it has been shown that schizophrenia and BD might share common genetic underpinnings [8]. Indeed, there are studies showing familial co-aggregation of schizophrenia and BD [9], common cognitive and neurostructural endophenotypes [10–12], and genetic variability [13].

Apart from genetic risk factors, it has been demonstrated that a number of environmental exposures including urban upbringing, stressful life events and early life stress, prenatal infections and obstetric complications interfering with brain development, and substance abuse or dependence may underlie the development of schizophrenia and BD [14, 15]. In light of several genetic and environmental factors involved in the etiology of these disorders, gene × environment (G × E) interactions have emerged as a novel research paradigm that might serve as a missing link in trajectories leading to schizophrenia or BD. These interactions refer to various scenarios, where genotype expression depends on exposure to a particular environment or in other words—the effects of environmental exposure depend on a particular genotype [16–18]. This approach has also indicated that the effects of some candidate genes might be significant only when certain environmental factors are taken into account.

To date, several G × E interactions have been reported in schizophrenia and BD; however, a recent systematic review of studies in this field was performed in 2013 and was limited to studies on patients with schizophrenia spectrum phenotypes [19]. Importantly, this systematic review focused on studies that examined psychotic disorders or various subgroups of clinically relevant or subthreshold psychotic symptoms as outcomes of G × E interactions. Therefore, studies investigating other outcome variables, such as cognitive performance or structural and functional brain alterations were not included. In turn, a systematic review of studies addressing G × E interactions in BD has not been performed so far. Therefore, the aim of this study was to perform an updated systematic and comprehensive review of studies investigating interactions between genetic variation in candidate genes and environmental factors in patients with schizophrenia spectrum phenotypes and BD.

## Materials and Methods

Search strategy followed PRISMA guidelines [20], although our systematic review was not registered. Three people (B.M., F.S., and D.F.) independently performed an online search for relevant publications in the PubMed, MEDLINE, ERIC (Education Resource Information Center), CINAHL, and Complete, Academic Search Complete and Health Source - Consumer Edition databases, using the following combination of keywords: (1) “schizophrenia” or “psychosis” or “bipolar disorder,” (2) “gene,” and (3) “environment.” In addition, our search strategy was supplemented by reference lists of relevant publications. After that, search results were compared and studies investigating interactions between candidate gene polymorphisms and environmental exposures in patients with schizophrenia spectrum phenotypes and BD were included in further analysis. There were following exclusion criteria: (1) publications written in non-English language; (2) non-original articles (commentaries, editorials, hypotheses, study protocols, methodological articles, reviews) and meta-analyses; (3) conference proceedings; (4) publications from studies with proxy measures of genetic liability, e.g., studies on relatives of patients with schizophrenia or BD and twin studies; (5) studies without genetic and/or environmental measures; (6) studies investigating DNA methylation patterns without genetic and/or environmental measures; (7) publications from GWASs; and (8) publications from studies on animal models and/or cell lines. We did not include studies investigating subclinical symptoms of BD in non-clinical samples, because these symptoms might be also closely related to major depressive disorder. Our systematic review covered publication records from database inception until 13 November 2016.

## Results

**Fig. 1:**
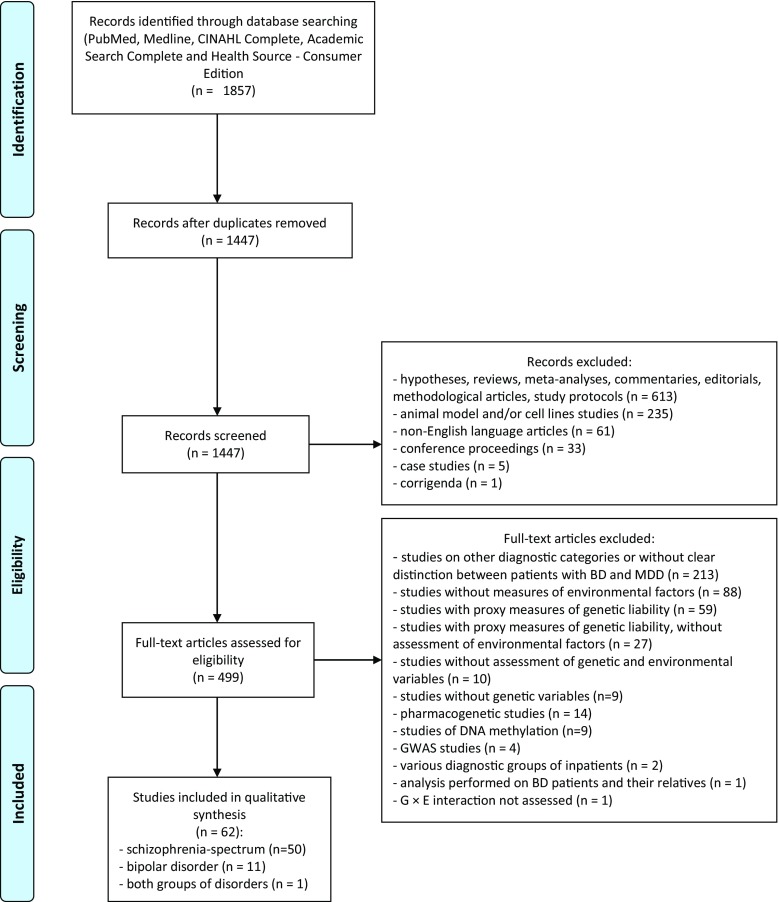
Selection of studies for systematic review based on PRISMA guidelines [20]

We identified 11 eligible studies performed on patients with BD and 50 studies on schizophrenia spectrum phenotypes as well as 1 study from both diagnostic groups (Fig. 1). These studies were grouped into five distinct clusters, based on environmental factors: (1) gene × cannabis interactions, (2) gene × stress and childhood trauma interactions, (3) gene × season of birth interactions, (4) gene × infectious factors interactions, and (5) gene × obstetric complications interactions (for overview of studies, see Tables 1 and 2).

**Table 1:** Studies investigating interactions between candidate genes and environmental factors in schizophrenia-spectrum phenotypes

Study (year)	Sample	Candidate gene polymorphisms	Candidate environmental factors	Outcome	Main results
De Castro-Catala et al. 2016 [21]	Two independent non-clinical samples of students (*n* = 808) and female twins (*n* = 621)	*BDNF* Val66Met (rs6265)	Childhood trauma (CTQ)	Subclinical psychotic experiences (CAPE)	A history of childhood trauma was associated with both positive and negative psychotic experiences. In the sample of students, the Val allele, especially in males, was associated with higher vulnerability of the effects of childhood trauma on psychotic experiences, while in the group of female twins this association was driven by the Met allele.
Cristobal-Narvaez et al. 2016 [22]	206 non-clinical young adults	*FKBP5* (rs3800373, rs9296158, and rs1360780)	Childhood trauma (CECA.Q) and social stress appraisal (assessed by ESM [23])	Negative affect, social contact, and psychotic-like experiences assessed by ESM [23]	There was a significant effect of interaction between the FKBP5 risk haplotype and childhood bullying on positive psychotic-like experiences, paranoia, and negative affect. The childhood bullying × the *FKBP5* haplotype interaction moderated the association of social stress appraisal with psychotic-like experiences and negative affect in daily life. Specifically, the associations were significantly increased in those with the risk haplotype, but not in individuals without the risk haplotype.
Gattere et al. 2016 [24]	124 individuals with early PD, 36 ARMS subjects, and 62 HCs	*BDNF* Val66Met (rs6265)	Stressful life events during previous 6 months (SRRS), perceived stress (PSS), and physical activity (IPAQ-SF)	Dietary patterns (FCQ-S)	Perceived stress was not associated with calorie intake in HCs. ARMS subjects with the Met allele and low perceived stress presented with increased caloric intake, while those with high perceived stress presented with decreased caloric intake. In patients with early psychosis, perceived stress was not associated with calorie intake. Perceived stress was associated with food craving in patients with psychosis. A similar association was present in ARMS subjects and HCs who were Val/Val homozygotes.
Mandelli et al. 2016 [25]	94 patients with SZ 176 HCs	*ST8SIA2* (rs3759917, rs11632521, rs3784722, rs4777989, rs2290492, rs8035760, rs11853992, and rs17522085)	Stressful life events (authors’ own questionnaire)	Age of psychosis onset	No significant interactions between the *ST8SIA2* gene polymorphisms and stressful life events on age of psychosis onset.
Nieman et al. 2016 [26]	147 ARMS subjects	*COMT* Val158Met (rs4680)	Cannabis use (CIDI)	Severity of ARMS psychopathology (CAARMS)	Weekly cannabis users at some point before the study had higher levels of positive symptomatology. This effect was stronger in the Val allele carriers and even more stronger in the Val/Val homozygotes.
Ursini et al. 2016 [27]	244 HCs, 162 patients with SZ, 140 siblings of patients with SZ and 214 parents of SZ patients	*BDNF* Val66Met (rs6265)—genotype, DNA methylation and expression	Obstetric complications—McNeil-Sjöström Scale [28]	Working memory—n-back task, dorsolateral prefrontal cortex activity, and schizophrenia risk	In Val/Val HCs, increased methylation at the rs6265 polymorphic site was associated with exposure to hypoxia-related early life events and working memory impairment (opposite effect was observed in Val/Met heterozygotes). The same effect was found for working memory-related prefrontal activity. Higher methylation levels in Val/Val homozygotes was associated with schizophrenia.
Colizzi et al. 2015 [29]	272 FEP patients and 234 HCs (case-control study), and 252 healthy subjects	*DRD2* (rs1076560)	Cannabis use (CEQ) in all participants	Psychosis risk, schizotypy (SPQ), and cognition (n-back working memory task) in healthy subjects	In cannabis users, T allele carriers had a threefold increase in psychosis risk compared to GG homozygotes. In daily users, T allele carriers had a fivefold increase in psychosis risk compared to GG homozygotes. In healthy subjects, daily users with T allele had higher schizotypy compared to cannabis-naïve T allele carriers, cannabis users with GG genotype and cannabis-naïve GG homozygotes. Cannabis users with T allele had lower working memory performance in comparison with other groups.
Ermis et al. 2015 [30]	80 male patients with SZ	*COMT* Val158Met (rs4680)	Cannabis use (disease history, family interviews and medical records)	Schizophrenia psychopathology (PANSS)	The Val/Val genotype was significantly more frequent in patients with premorbid cannabis use compared to those without cannabis use. There were higher levels of positive and negative symptoms in Val/Val homozygotes compared to the Met allele carriers.
Green et al. 2015 [31]	444 patients with SZ and 292 HCs	*FKBP5* (rs1360780, rs9470080, rs4713902, and rs9394309)	Childhood trauma (CAQ)	Cognitive performance (RBANS, premorbid IQ—WTAR, the letter number sequencing—WAIS, COWAT)	There were significant main effects of the rs1360870 genotype and childhood trauma and a significant interaction between these variables affecting attention in both groups (CC homozygotes performed worse in the context of childhood trauma). Additionally, there were significant main effects of this polymorphism on global cognition in SZ patients (TT homozygotes performed worse).
Wegelius et al. 2015 [32]	457 subjects from the Finnish Schizophrenia Birth Cohort	*NDE1* (rs4781678, rs2242549, rs881803, and rs2075512), *PDE4B* (rs7412571) *PDE4D* (haplotype)	Birth weight	A diagnosis of SZ (medical records, SCID)	High birth weight was associated with schizophrenia risk in subjects homozygous for risk alleles (a four-SNP haplotype spanning *NDE1* gene and one of its constituent SNPs—rs4781678).
Aas et al. 2014 [33]	182 patients with SZ spectrum diagnosis, 130 patients with BD, 11 patients with MDD with psychotic features	*BDNF* Val66Met (rs6265)	Childhood trauma (CTQ)	Hippocampal subfield measures and BDNF mRNA levels	Met allele carriers with high levels of childhood trauma had significantly lower levels of BDNF mRNA and reduced CA2/3 and CA4 subfields of dentate gyrus.
Ajnakina et al. 2014 [34]	291 FEP patients and 218 HCs	*FKBP5* (rs1360780)	Cannabis use (CEQ) Childhood trauma (CECA.Q)	FEP—risk of psychosis (ICD-10)	The *FKBP5* rs1360780 polymorphism was associated with the risk of psychosis only after adjustment for environmental factors. There was a significant effect of the interaction between the *FKBP5* rs1360780 polymorphism and parental separation on psychosis risk.
Hernaus et al. 2014 [35]	89 patients with PD and 95 healthy siblings	*FKBP5* (rs9296158, rs4713916, rs992105, and rs38003), *BDNF* Val66Met (rs6265)	Childhood trauma (CTQ)	Hippocampal volume and cognitive performance (auditory verbal learning task and block design task from WAIS)	There were no significant effects of interactions between studied polymorphism and childhood trauma on hippocampal volumes and cognition.
McCarthy-Jones et al. 2014 [36]	333 SZ spectrum patients	*FOXP2* (rs1456031, rs2396753, and rs2253478)	Childhood trauma (CTQ)	Lifetime history of AVHs (DIP)	There was a significant effect of the interaction between the rs1456031 polymorphism and parental emotional abuse. Emotional abuse was only associated with significantly higher levels of AVHs in patients with CC genotype. However, in the absence of emotional abuse TT homozygotes had significantly higher levels of AVHs than those with CC genotype.
Pishva et al. 2014 [37]	Sample I: 112 HCs Sample II: 434 general population twins Sample III: 85 siblings of patients with PD Sample IV: 110 patients with PD Sample V: 126 patients with at least one episode of MDD	31 SNPs in epigenetic-regulatory genes: *MTHFR*, *DNMT1*, *DNMT3A*, and *DNMT3B*	Daily life stressors (ESM)	Emotional responses (ESM)	Three SNPs in the *DNMT3A* gene (rs11683424, rs1465764, rs1465825) and the *MTHFR* rs1801131 moderated the effect of stressful events on negative affect. Effects of the *DNMT3A* rs11683424 polymorphism were consistent in the majority of samples.
Collip et al. 2013 [38]	401 general population twins, 195 patients with psychosis, 200 unaffected siblings and 175 HCs	*FKBP5* (rs9296158, rs4713916, rs1043805, and rs1360780)	Childhood trauma (CTQ)	Salivary free cortisol levels Psychotic experiences (CAPE, SIS-R)	There was a significant interaction between the rs9296158/rs4713916 polymorphisms and childhood trauma on psychotic symptoms and cortisol levels in the twin sample. Similar findings were obtained for the rs4713916 polymorphism in siblings and for the rs9296158 polymorphism in patients. Specifically, the A allele carriers at both polymorphisms were most vulnerable to childhood trauma.
De Sousa et al. 2013 [39]	403 patients with SZ and schizoaffective disorder	*COMT* polymorphisms (rs4680, rs4633, rs4818, and rs6269)	Cannabis use (DSM-IV, clinical assessment)	Age of psychosis onset	There were no significant interactions between the *COMT* gene polymorphisms and age of psychosis onset.
Onwuaemeze et al. 2013 [40]	235 SZ patients	*MAPK14* polymorphisms (rs3804454, rs2237094, rs12199654, rs851007, rs851006, rs3804452, rs8510, rs7757672, and rs916346) and *CNR1* rs12720071 polymorphism	Substance use (CASH, DSM-IV criteria)	White matter brain volumes	The rs12199654 AA homozygotes with cannabis abuse/dependence had significantly smaller total cerebral and lobar white matter volumes. This effect remained significant after controlling for the *CNR1* genotype. There were significant main effects of the *MAPK14/CNR1* diplotype and the interaction between this diplotype and cannabis abuse/dependence on white matter brain volumes. The effects of these two polymorphisms were additive.
Ramsay et al. 2013 [41]	237 general population individuals	*COMT* Val158Met (rs4680) *BDNF* Val66Met (rs6265)	Childhood trauma (K-SADS)	Psychotic experiences (K-SADS)	Individuals with the *COMT* Val/Val genotype exposed to childhood trauma were more likely to report psychotic experiences than those with other *COMT* genotypes (trend level significance). No significant interactions with childhood trauma were found for the *BDNF* Val66Met polymorphism.
Vinkers et al. 2013 [42]	Discovery sample: 918 (general population) Replication sample: 339 healthy controls and healthy siblings of patients with NAPD	*COMT* Val158Met (rs4680)	Cannabis use (CIDI, the monetary amount spent on cannabis) Childhood trauma (CTQ)	Psychotic experiences (CAPE)	Val/Val homozygotes from the discovery sample exposed to cannabis use and childhood maltreatment had significantly higher levels of psychotic experiences compared to Met allele carriers (Val/Met and Met/Met genotypes). These results did not reach statistical significance in the replication sample.
Bhattacharyya et al. 2012 [43]	35 HCs	*DAT1* 3′UTR VNTR, *AKT1* rs1130233	Cannabis (delta-9-THC) intake	Delta-9-THC-induced psychotic experiences (PANSS)	The GG homozygotes for the *AKT1* rs1130233 polymorphism and also carriers of the 9-repeat allele of the *DAT1* 3′UTR VNTR had greater increase in psychotic symptoms induced by delta-9-THC compared to subjects with other genotypes.
Di Forti et al. 2012 [44]	489 FEP patients and 278 HCs	*AKT1* rs2494732	Cannabis use (lifetime use and frequency of use)	FEP—risk of psychosis (ICD-10)	The CC homozygotes with a history of cannabis use showed a greater than twofold increase in the likelihood of PD in comparison with the TT homozygotes. Among daily cannabis users, individuals with the CC genotype demonstrated a sevenfold increase in the odds of psychosis compared to the TT homozygotes.
Husted et al. 2012 [45]	162 patients with SZ spectrum disorders and 75 HCs	*CAPON* (*NOS1AP*) rs12742393	Cannabis use, childhood trauma (SCID-I)	SZ risk (SCID-I)	No significant effects of interactions between the *NOS1AP* genotype and environmental factors on SZ risk were found.
Peerboms et al. 2012 [46]	84 patients with NAPD and 107 HCs	*COMT* Val158Met (rs4680), *MTHFR* C677T (rs1801133), *MTHFR* A1298C (rs1801131)	Daily life stressors (ESM)	Psychotic experiences (ESM self-report) in NAPD (OPCRIT or CASH)	Patients with the *MTHFR* T allele, *COMT* Met/Met homozygotes showed the largest increases in psychotic experiences in response to stress. In patients who were the *MTHFR* CC homozygotes, there was no interaction between the *COMT* Val158Met polymorphism and stress. There was no moderating effect of *MTHFR* A1298C on the interaction between the *COMT* Val158Met polymorphism and stress.
Alemany et al. 2011 [47]	533 HCs	*BDNF* Val66Met (rs6265)	Stress-childhood adversity defined as childhood neglect and childhood abuse (CTQ)	Psychotic-like experiences (CAPE)	Individuals carrying the Met allele had higher scores on adult positive psychotic-like experiences when childhood abuse was present, when compared to Val/Val homozygotes. No significant gene × environment interaction was detected with respect to childhood neglect.
Collip et al. 2011 [48]	86 patients with NAPD and 109 HCs	*COMT* Val158Met (rs4680)	Daily life stressors (ESM)	Psychotic experiences (ESM self-report) in NAPD (OPCRIT or CASH)	Patients being the *COMT* Met/Met homozygotes showed increased psychotic reactivity to stress compared to the Val allele carriers.
Costas et al. 2011 [49]	748 patients with SZ recruited in two independent samples	*COMT* (rs737865, rs6269, rs4633, rs4818, and rs4680)	Lifetime cannabis use according to DSM-IV criteria or medical records	The risk of cannabis use in patients with schizophrenia	Patients with low activity variants were significantly more prone to use cannabis (joint analysis, results were consistent between two independent samples). In the joint analysis, the probability of lifetime cannabis use was twofold higher in the rs4680 Met/Met homozygotes compared to Val/Val homozygotes.
Decoster et al. 2011 [50]	585 patients with SZ	*BDNF* Val66Met (rs6265)	Substance use (CIDI)	Age of psychosis onset	In female patients, cannabis use was associated with earlier age of psychosis onset in the Met allele carriers, but not in Val/Val homozygotes. In male patients, cannabis use was associated with earlier age of psychosis onset, regardless of the *BDNF* genotype. The main effect of the *BDNF* genotype on age of psychosis onset was not significant in the whole group as well as in males and females separately.
Demontis et al. 2011 [51]	Three independent samples of SZ patients and HCs (Denmark I: 385 patients and 780 HCs, Denmark II: 365 patients and 434 HCs, Denmark III: 234 patients and 286 HCs)	81 SNPs in *GRIN2A* and *GRIN2B* genes	Maternal HSV-2 seropositivity	The risk of schizophrenia (ICD-10)	The *GRIN2B* rs1806194 polymorphism was significantly associated with schizophrenia risk after Bonferroni correction. There were significant effects of interactions between two SNPs in the *GRIN2B* gene (rs1805539 and rs1806205) and maternal HSV-2 seropositivity on schizophrenia risk after Bonferroni correction.
Estrada et al. 2011 [52]	80 patients with SZ spectrum disorders and 77 patients with non-psychotic disorders (conduct and affective disorders)	*COMT* Val158Met (rs4680)	Cannabis use (DIGS and urine drug scrrening)	Age at onset of psychiatric disorders	There were no significant differences in genotype distributions between diagnostic groups or between cannabis users or non-users. However, the Val/Val homozygotes had earlier age of psychosis onset compared to the Met allele carriers. This effect was not significant in patients with non-psychotic disorders.
Ho et al. 2011 [53]	235 patients with SZ	12 SNPs in the *CNR1* gene	Substance use (CASH)	Brain volumes and cognitive performance (WAIS-R, WCST, TMT-A, and Shipley Institute of Living Scale abstractions subtest)	There were significant main effects of the *CNR1* gene SNPs (rs7766029, rs12720071, and rs9450898) on white matter volumes. Patients with cannabis abuse/dependence had smaller frontotemporal white matter volumes. There were significant effects of the interaction between the rs12720071 genotype and cannabis abuse/dependence on parietal white matter volumes and cognition (problem solving).
Muntjewerff et al. 2011 [54]	742 patients with SZ	*MTHFR* C677T (rs1801133)	Seasonality of birth (winter birth)	SZ (CASH)	There was no evidence for the interaction between *MTHFR* 677TT genotype and winter birth in the development of SZ.
Van Winkel et al. 2011 [55]	801 patients with NAPD, 740 unaffected siblings and 419 HCs	152 SNPs in 42 genes	Cannabis (recent use —urinary result, lifetime pattern of use—CIDI)	Non-affective psychotic disorder Positive schizotypy (factor structure of SIS-R)	Case-only design: the *AKT1* rs2494732 polymorphism showed association with lifetime use, restricted to use preceding onset of psychosis. Case-siblings design: *AKT1* rs2494732, *AKT1* rs1130233, and *LRRTM1* rs673871 showed significant interactions with recent cannabis use on positive schizotypy. In follow-up, patients with the *AKT1* rs2494732 CC genotype displayed approximately twofold higher odds of being diagnosed with PD when having used cannabis in comparison with TT homozygotes. Case-control design: no significant interaction was shown.
Haukvik et al. 2010 [56]	54 schizophrenia patients and 53 HCs	*BDNF*, *DTNBP1*, *GRM3*, and *NRG1* (32 SNPs)	Obstetric complications assessed with McNeil-Sjöström Scale [28]	Hippocampal volume	Severe obstetric complications were associated with reduced hippocampal volumes in both groups. There were no significant main effects of studied SNPs on hippocampal volumes. There was a significant effect of the interaction between the *GRM3* rs13242038 polymorphism and severe obstetric complications on hippocampal volumes in patients with schizophrenia and HCs.
Pelayo-Teran et al. 2010 [57]	169 FEP patients	*COMT* Val158Met (rs4680)	Cannabis use in the previous year (clinical assessment)	Age of onset, DUP	Cannabis users had significantly earlier age of psychosis onset. There was significant interaction between *COMT* genotype and cannabis use on age of psychosis onset and DUP. Post hoc analyses revealed that the effect of *COMT* genotype on age of psychosis onset was significant only in cannabis non-users (longer DUP and earlier age of psychosis onset in Val/Val homozygotes.
Zammit et al. 2010 [58]	2630 HCs	*COMT* Val158Met (rs4680), rs4818, rs6269, rs737865, rs2097603, rs165599	Cannabis (self-report postal questionnaires completed at age 14)	Psychotic experiences (incident psychotic experiences at age 16)	There was no evidence of an interaction between *COMT* SNPs and cumulative use of cannabis on the development of psychotic experiences.
Gutierrez et al. 2009 [59]	91 patients with SZ and 192 HCs	*COMT* Val158Met (rs4680)	Cannabis use (frequency of taking in the previous month)	Schizophrenia risk (DSM-IV)	Cannabis consumption was significantly more frequent in the group of patients. In the Val allele carriers, cannabis consumption rates were higher in female schizophrenia patients compared to healthy women, while in the Met/Met homozygotes, cannabis consumption rates were higher in healthy women compared to female schizophrenia patients (non-significant interaction).
Henquet et al. 2009 [60]	31 patients with PD and 25 HCs	*COMT* Val158Met (rs4680)	Cannabis use (ESM self-report)	Psychosis liability (CAPE) Psychotic experiences (ESM self-report)	Cannabis significantly increased hallucinatory experiences only in individuals who were carriers of the Val allele and also had high levels of psychometric psychosis liability. No such associations were observed for delusional experiences.
Kantrowitz et al. 2009 [61]	92 patients with PD	*COMT* Val158Met (rs4680)	Adolescent cannabis use prior to age 18 (SCID)	Psychotic disorder (SCID)	No differences in *COMT* Val58Met genotype distribution with respect to adolescent cannabis use neither in Caucasian nor in African-American patients with psychotic disorder.
Keri et al. 2009 [62]	200 SZ	*NRG1* (rs6994992, rs10954867, and rs7005288)	Psychosocial stress (conflict-related family interactions—PSP)	Odd and unusual thought content during neutral and conflict-related family interactions over the two 10-min interactions with one of the family members: mothers, fathers, wives, husbands, and siblings in SZ (MINI)	Patients with the *NRG1* TT genotype showed more unusual thoughts during conflict-related interactions than patients with CT and CC genotypes (rs6994992). There were no significant differences between the *NRG1* CT and CC patients. There were no significant differences among patients with different NRG1 genotypes during neutral interactions.
Simons et al. 2009 [63]	579 HC female twins	*COMT* Val158Met (rs4680) *BDNF* Val66Met (rs6265)	Event stress and social stress in daily life (ESM)	Feelings of paranoia (ESM self-report)	Carriers of the *COMT* 158 Val allele displayed more feelings of paranoia in response to event stress compared to Met carriers. Carriers of the *BDNF* 66Met allele showed more social stress-induced paranoia than individuals with the Val/Val genotype.
Nicodemus et al. 2008 [64]	116 SZ spectrum disorders and 134 HCs	*AKT1*, *BDNF*, *CAPON* (*NOS1AP*), *CHRNA7*, *COMT*, *DTNBP1*, *GAD1*, *GRM3*, *NOTCH4*, *NRG1*, *PRODH*, *RGS4*, *TNF-α*	Obstetric complications (questionnaires were completed by parents of affected individuals and of control subjects)	SZ spectrum disorders (SCID-I, SCID-II)	Probands with obstetric complications were more likely to have minor allele at the *AKT1* rs2494735 and rs1130233 polymorphisms, major allele at the *BDNF* rs2049046 polymorphism and the minor allele at the rs76882600 polymorphism, minor allele at the *DTNBP1* rs875462, minor allele at the *GRM3* rs7808623.
Van Winkel et al. 2008 [65]	31 patients with PD and cannabis use, 25 non-psychotic cannabis users	*COMT* Val158Met (rs4680)	Daily life stressors (ESM)	Psychotic experiences (ESM self-report)	Subjects with the *COMT* 158 Met/Met genotype had greater increase in overall psychotic experiences in response to daily stressors in comparison to Val/Met and Val/Val carriers both among patients and healthy controls.
Shirts et al. 2007 [66]	Three independent samples of SZ patients (primary sample: 236 patients and 240 HCs, Baltimore: 272 cases and 108 HC, Pittsburgh: 221 case-parent trios)	26 SNPs from the locus 6p21	CMV and HSV1 seropositivity	Schizophrenia risk (DSM-IV)	In Baltimore controls, the *MICB* rs1051788 polymorphism was associated with HSV1 seropositivity, while the *MICB* rs2523651 polymorphism was associated with CMV seropositivity. The former association was also observed in Pittsburg parents. None of them was observed in patients with schizophrenia. There was a significant transmission distortion of the *MICB* SNPs (rs1051788 and rs1055569) in case-parent trios regardless of antibody status. The association between the *MICB* rs1051788 polymorphism and schizophrenia risk was not significant.
Stefanis et al. 2007 [67]	306 male HCs	*COMT* Val158Met (rs4680)	Stress (recruitment in the army)	Psychotic symptoms (SCL-90-R)	Carriers of the *COMT* 158Val allele were more sensitive to psychosis inducing effects of stress exposure at army in comparison with Met/Met homozygotes.
Zammit et al. 2007 [68]	750 patients with SZ and 688 HCs	*CNR1* rs1049353, *COMT* Val158Met (rs4680), rs737865, and rs165599 *CHRNA7*-86C/T	Cannabis use (interview and case-note records)	Schizophrenia (SCAN, OPCRIT)	No evidence of interaction between cannabis use and selected polymorphisms.
Henquet et al. 2006 [69]	30 patients with PD, 12 first and second-degree relatives (3 with BPD and 1 with MDD) and 32 HCs	*COMT* Val158Met (rs4680)	Cannabis (delta-9-THC) intake	Psychosis liability (CAPE) Delta-9-THC-induced psychotic experiences (PANSS)	The *COMT* Val/Val homozygotes had largest increase in delta-9-THC -induced psychotic experiences varying as a function of psychometric psychosis liability.
Caspi et al. 2005 [70]	803 HCs	*COMT* Val158Met (rs4680)	Cannabis (follow-ups carried out at ages 3, 5, 7, 9, 11, 13, 15, 18, 21, 26)	Psychosis outcomes assessed at age 26 (DIS): schizophrenia and schizophreniform disorder. A 60-item questionnaire was mailed to persons nominated by each study member at age 26 as “someone who knows you well”	Adolescent cannabis use was associated with increased risk of schizophrenia and schizophreniform disorder in adulthood in the *COMT* 158Val allele carriers, but not in Met/Met homozygotes.
Chotai et al. 2003 [71]	147 patients with SZ and 395 HCs	*TPH1* A218C (rs1800532) *5-HTTLPR* *DRD4*	Seasonality of birth	SZ (OPCRIT)	The frequency of the DRD4 7-repeat allele showed one-cyclic season of birth variation in women with SZ.
Tochigi et al. 2002 [72]	110 patients with SZ and 493 HCs	*HLA* (*HLA-A24*, *HLA-A26*)	Seasonality of birth (winter birth)	SZ (DSM-IV)	No association between *HLA* (-A24 or -A26) and winter birth (December–March) in patients with schizophrenia.
Narita et al. 2000 [73]	60 SZ patients with HLA-DR1 and 307 SZ patients without HLA-DR1	*HLA* (*HLA-DR1*)	Seasonality of birth (winter birth)	SZ (DSM-IV)	Increased incidence of winter births (February–March) in patients with *HLA-DR1* than in patients without *HLA-DR1*.

*AKT1* RAC-α serine/threonine-protein kinase, *ARMS* At-Risk Mental State, *AVHs* auditory verbal hallucinations, *BD* bipolar disorder, *BDNF* brain-derived neurotrophic factor, *CAARMS* Comprehensive Assessment of At-Risk Mental State [74], *CAPE* Community Assessment of Psychic Experiences [75], *CAPON* (*NOS1AP*) nitric oxide synthase 1 adaptor protein, *CAQ* Childhood Adversity Questionnaire [76], *CASH* Comprehensive Assessment of Symptoms and History [77], *CECA*.*Q* the Childhood Experience and Abuse Questionnaire [78], *CEQ* Cannabis Experience Questionnaire [79], *CHRNA7* neuronal acetylcholine receptor subunit alpha-7, *CIDI* the Composite International Diagnostic Interview [80], *CMV* cytomegalovirus, *CNR1* cannabinoid receptor 1, *COMT* catechol-*O*-methyltransferase, *COWAT* the Controlled Oral Word Association Test [81], *CTQ* Childhood Trauma Questionnaire [82], *DAT1* dopamine active transporter 1, *DIP* Diagnostic Interview for Psychoses [83], *DIS* Diagnostic Interview Schedule for DSM-IV [84], *DNMT* DNA methyltransferase, *DRD4* dopamine D4 receptor, *DTNBP1* dystrobrevin binding protein 1, *ESM* experience sampling methodology, *FCQ*-*S* Food Craving Questionnaire-State [85], *FEP* first-episode psychosis, *FKBP5* FK506 binding protein 5, *FOXP2* forkhead box protein 2, *GAD1* glutamate decarboxylase 1, *GRIN2A* glutamate ionotropic receptor NMDA type subunit 2A, *GRIN2B* glutamate ionotropic receptor NMDA type subunit 2B, *GRM3* glutamate metabotropic receptor 3, *HCs* healthy controls, *HLA* human leukocyte antigen, *HSV*-*1* Herpes Simplex Virus 1, *IPAQ*-*SF* International Physical Activity Questionnaire Short Form [86], *K*-*SADS* the Schedule for Affective Disorders and Schizophrenia for School-Aged Children [87], *MAPK14* mitogen-activated protein kinase 14, *MDD* major depressive disorder, *MINI* Mini-International Neuropsychiatric Interview [88], *MTHFR* methylenetetrahydrofolate reductase, *NAPD* non-affective psychotic disorder, *NDE1* nuclear distribution protein nudE homolog 1, *NOS* not otherwise specified, *NOTCH4* neurogenic locus notch homolog 4, *PANSS* Positive and Negative Syndrome Scale [89], *NRG1* neuregulin 1, *OPCRIT* the Operational Criteria for Psychotic Illness Checklist [90], *PANSS* the Positive and Negative Syndrome Scale [89], *PD* psychotic disorder, *PRODH* proline dehydrogenase 1, *PSP* Patient Symptom Profile [91, 92], *PSS* Perceived Stress Scale [93], *RBANS* Repeatable Battery for the Assessment of Neuropsychological Status [94], *RGS4* regulator of G protein signaling 4, *SCID* Structured Clinical Interview for DSM-IV [95], *SIS*-*R* the Structured Interview for Schizotypy-Revisited [96], *SNPs* single nucleotide polymorphisms, *SPQ* Schizotypal Personality Questionnaire [97], *SRRS* Holmes-Rahe Social Readjustment Rating Scale [98], *ST8SIA2* ST8 alpha-*N*-acetyl-neuraminide alpha-2,8-sialyltransferase 2, *SZ* schizophrenia, *TNF*-*α* tumor necrosis factor-α, *TPH1* tryptophan hydroxylase 1, *WAIS* Wechsler Adult Intelligence Scale [99], 5-*HTTLPR* serotonin-transporter-linked polymorphic region

**Table 2:** Studies investigating interactions between candidate genes and environmental factors in bipolar disorder

Study (year)	Sample	Candidate gene polymorphisms	Candidate environmental factors	Outcome	Main results
Chotai et al. 2003 [71]	456 patients with BD 351 patients with MDD 147 patients with SZ 395 HCs	*TPH1* A218C (rs1800532), *5-HTTLPR* L/S (rs25531), and *DRD4* 7-repeat allele	Season of birth	The risk of BD, MDD, SZ	The allele frequencies did not show any significant variation with respect to seasons defined as four 3-month periods beginning in January. However, the analysis of one-cyclic month of birth variations showed that the *TPH1* allele A had a positive peak around the birth month December and a negative peak around June in men with BD, but not in women with BD. There were more cases of BP among men with *TPH1* allele A born in Nov-Jan, and less cases of BP among women with *TPH1* allele A born in Feb-Jul in comparison to healthy controls. Moreover, analysis of two cycles per year showed differences in the *DRD4* gene variations both among women and men with BD.
Dickerson et al. 2006 [100]	107 patients with BD 95 HCs	*COMT* Val158Met (rs4680)	Antibodies to HSV-1	Cognitive performance (RBANSS)	The *COMT* 158Val/Val genotype and HSV-1 seropositivity were independent predictors of lower global cognitive performance in patients with BD but not in HCs. Patients with both *COMT* 158Val/Val genotype and HSV-1 seropositivity were 85 times more likely to be in the lowest quintile of global cognitive performance.
De Pradier et al. 2010 [101]	137 patients with BD	*5-HTTLPR* L/S (rs25531)	Cannabis abuse or dependence (DIGS) Childhood trauma (THQ)	Lifetime occurrence of psychotic symptoms (DIGS)	The interaction between the S allele and childhood sexual abuse increased odds of cannabis abuse or dependence. Cannabis abuse or dependence and S allele, but not childhood sexual abuse, were significantly more frequent in those patients with lifetime occurrence of psychotic symptoms.
Hosang et al. 2010 [102]	487 patients with BD type 1 598 HCs	*BDNF* Val66Met (rs6265)	Stressful life events (LTE-Q)	Worst episodes of depression and mania (SCAN)	The *BDNF* 66Met allele carriers with higher levels of stressful life events had higher severity of the worst depression ever.
Savitz et al. 2010 [103]	222 patients with BD type 1	*DRD4* 48 bp VNTR, *DRD2* Taq1A, *DAT1* 3′VNTR, and *MAOA* promoter VNTR	Childhood trauma (CTQ)	Schizotypy (STA)	The *COMT* 158Val allele was associated with higher levels of schizotypy in patients exposed to higher levels of childhood trauma. There were no main effects of the *COMT* 158Val/Met polymorphism on the levels of schizotypy.
Debnath et al. 2013 [104]	561 patients with BD 161 HCs	*HLA-G* 14 bp ins/del (66554220)	Season of birth	The risk of BD	The *HLA-G* ins/ins genotype was significantly less frequent in patients with BD. The prevalence of this genotype was significantly lower in patients born in the winter season.
Miller et al. 2013 [105]	80 patients with BD (43 with type I, 33 with type II, 4 with NOS)	*BDNF* 66Val/Met (rs6265)	Childhood trauma (CTQ)	Severity and chronicity of BD (CGI-BP-OS)	The *BDNF* 66Met allele carriers with a history of childhood sexual abuse had 21% higher levels of BD severity and chronicity as well as 35% earlier age of BD onset compared to those without this type of childhood trauma. This interaction was not significant in regression analysis
Bortolasci et al. 2014 [106]	45 patients with BD 91 patients with MDD 199 HCs	*PON1* Q192R (rs662)	Cigarette smoking	The risk of BD and MDD	The interaction between *PON1* QQ genotype and cigarette smoking increased the risk of BD and MDD in separate analyses.
Breen et al. 2015 [107]	631 patients with BD and lifetime history of suicide attempts 657 patients with BD without lifetime history of suicide attempts	235 HPA axis SNPs	Childhood physical and sexual abuse (ELES)	Suicide attempts (DIGS)	No significant effects of interactions between polymorphisms in HPA axis genes and childhood trauma on lifetime occurrence of suicide attempts.
Oliveira et al. 2015 [108]	531 patients with BD (391 with type 1, 113 with type 2 and 27 with NOS)	*TLR2* (rs4696480 and rs3804099) and *TLR4* (rs1927914 and rs11536891)	Childhood trauma (CTQ)	Age of BD onset	Patients with the *TLR2* rs3804099 TT genotype and a history of sexual abuse had earlier age of BD onset compared to other patients in Kaplan-Meier survival curve analysis but not in regression analysis.
Zeni et al. 2016 [109]	29 children and adolescents with BD 22 HCs	*BDNF* Val66Met (rs6265)	Family functioning (FES)	Hippocampal volumes	A significant interaction between Met allele and low scores of cohesion subscale (from Family Environment Scale-Revised) on the left hippocampal volume in patients with BD. Main effects were not significant. There were no significant differences between HCs and BD patients in terms of hippocampal volume.
Oliveira et al. 2016 [110]	138 patients with BD 167 HCs	*TLR2* (rs4696480 and rs3804099), *TLR4* (rs1927914 and rs11536891) and *NOD2* (rs2066842)	*Toxoplasma gondii* seropositivity	The risk of BD	A trend toward statistical significance for the interaction between Toxoplasma gondii seropositivity and the *TLR2* rs3804099 polymorphism in conferring BD risk.

*BD* bipolar disorder, *BDHI* Buss-Durkee Hostility Inventory [111], *BDNF* brain-derived neurotrophic factor, *BGAI* Brown-Goodwin Aggression Inventory [112], *BIS* Barratt Impulsivity Scale [113], *CGI*-*BP*-*OS* Clinical Global Impressions Bipolar Version Overall Severity of Illness [114], *COMT* catechol-*O*-methyltransferase, *CTQ* Childhood Trauma Questionnaire [82], *DAT1* dopamine active transporter 1,DIGS Diagnostic Interview for Genetic Studies [115], *DRD2* dopamine D2 receptor, *DRD4* dopamine D4 receptor, *ELES* Early Life Events Scale unpublished [107], *FES* Family Environment Scale [116], *HCs* healthy controls, *HDRS* Hamilton Depression Rating Scale [117], *HLA*-*G* histocompatibility antigen class I,G, *HPA* hypothalamic-pituitary-adrenal, *HSV*-*1* Herpes Simplex Virus 1, *LTE*-*Q* the List of Threatening Experiences Questionnaire [118], *MAOA* monoamine oxidase A, *MDD* major depressive disorder, *PBI* Parental Bonding Instrument [119], *PON1* paraoxonase 1, *SCAN* Schedules for Clinical Assessments in Neuropsychiatry [120], *STA* the Schizotypal Personality Scale [121], *SZ* schizophrenia, *THQ* Trauma History Questionnaire [122], *TLR2* toll-like receptor 2, *TLR*-*4* toll-like receptor 4, *TPH1* tryptophan hydroxylase 1, *VNTR* variable number of tandem repeats, *5*-*HTTLPR* serotonin-transporter-linked polymorphic region

### Gene × Cannabis Interactions

To date, interactions between cannabis use and genetic factors have been tested in 24 studies performed on patients with schizophrenia spectrum phenotypes [26, 29, 30, 34, 39, 40, 42–45, 49, 50, 52, 53, 55, 57–61, 68–70]. The vast majority of these studies analyzed the impact of dopaminergic genes. In one longitudinal study of a representative cohort followed from adolescence to adulthood, Caspi et al. [70] found that cannabis use in adolescence increased odds of schizophrenia and schizophreniform disorder in the catechol-*O*-methyltransferase (*COMT*) 158Val allele carriers, but not in the *COMT* 158Met allele homozygotes. Subsequent studies have provided mixed results. Henquet et al. [69] revealed that exposure to delta-9-tetrahydrocannabinol (THC) leads to the largest increase in psychotic symptoms and memory impairment in patients with psychotic disorders and healthy controls homozygous for the *COMT* 158Val allele. More specifically, the same group showed that carriers of the *COMT* 158Val allele, but not subjects with the *COMT* 158 Met/Met genotype, present an increase in hallucinations after cannabis exposure that is conditional on prior psychosis liability [60]. Similar results showing higher levels of positive psychopathology in the COMT 158Val allele carriers (or COMT 158 Val/Val homozygotes) have been reported in cannabis users among at-risk mental state (ARMS) individuals [26] and schizophrenia patients [30]. Interestingly, in one study [52], the effects of the interaction between the *COMT* Val158Met polymorphism and cannabis use on age at onset of schizophrenia spectrum disorders and non-psychotic disorders were tested. Authors found earlier age at onset in patients with schizophrenia spectrum disorders with the *COMT* 158 Val/Val genotype compared to the *COMT* 158Met allele carriers. This effect was insignificant in patients with non-psychotic disorders. A similar interaction between cannabis use and the *COMT* Val158Met polymorphism was reported by Pelayo-Teran et al. [57]. Authors found that earlier age of psychosis onset and longer duration of untreated psychosis in the *COMT* Val/Val first-episode psychosis (FEP) patients, who were cannabis non-users. This effect was insignificant in cannabis users with FEP. Vinkers et al. [42] also found in the discovery sample of general population individuals that the *COMT* 158Val/Val homozygotes exposed to cannabis use and childhood maltreatment have higher levels of psychotic experiences than carriers of the *COMT* 158Met allele. However, these findings were not replicated in the confirmation sample. In one study, the opposite results were obtained showing that the probability of lifetime cannabis use was twofold higher in schizophrenia patients with the *COMT* 158 Met/Met genotype compared to Val/Val homozygotes [49]. Other studies have not confirmed the interaction between the *COMT* 158Val/Met polymorphism and cannabis use on the risk of psychosis [55, 61, 68] or risk of subclinical psychotic experiences [58] and age of psychosis onset [39]. In one study, no significant differences in the frequency of cannabis use between schizophrenia patients with distinct *COMT* genotypes were found [59]. Additionally, Zammit et al. [68] did not find any significant interactions between variation in the cannabinoid receptor 1 (*CNR1*) gene and the cholinergic receptor, nicotinic, alpha 7 (*CHRNA7*) genes, and cannabis use. However, Ho et al. [53] found that schizophrenia patients with cannabis abuse/dependence with the *CNR1* rs12720071 G allele had lower parietal white matter volumes (trend level significance) and performed significantly worse on problem solving tasks. In agreement with these findings, a more recent study demonstrated an interaction between the *CNR1* rs12720071/*MAPK14* rs12199654 diplotype and cannabis abuse/dependence on white matter volumes in another sample of patients with schizophrenia [40].

In three studies [43, 44, 55], the interaction between genetic variation in the RAC-alpha serine/threonine-protein kinase 1 (*AKT1*) gene and cannabis use has been tested. Van Winkel et al. [55] addressed this issue in a study of patients with non-affective psychosis, unaffected siblings of patients with psychosis and healthy controls that initially included a set of 152 single nucleotide polymorphisms (SNPs) in 42 candidate genes. Main effects of three SNPs on positive schizotypy, after recent cannabis exposure, remained significant after correction for multiple testing in the group of unaffected siblings. These variants included two SNPs in the *AKT1* gene (rs2494732 and rs1130233) and one SNP in the leucine-rich repeat transmembrane protein 1 (LRRTM1) gene (rs673871). There were significant interactions between the *AKT1* rs2494732 polymorphism and lifetime or restricted cannabis use on psychotic symptoms in the subsequent case-only analysis. In the case-sibling design, patients with the *AKT1* rs2494732 CC genotype displayed approximately twofold higher odds of being diagnosed with psychotic disorder when having used cannabis in comparison with TT homozygotes. However, no significant interactions were found in the case-control design. Importantly, DiForti et al. [44] confirmed these findings for the *AKT1* rs2494732 gene polymorphism. The *AKT1* rs2494732 CC homozygotes with a history of cannabis use showed a greater than twofold increase in the likelihood of having a psychotic disorder in comparison with the *AKT1* rs2494732 TT homozygotes. Among daily cannabis users, individuals with the *AKT1* rs2494732 CC genotype demonstrated a sevenfold increase in the odds of developing psychosis compared to the *AKT1* rs2494732 TT homozygotes. Similarly, in the experimental study by Bhattacharyya et al. [43], healthy individuals with the *AKT1* rs1130233 GG genotype and the dopamine transporter (*DAT1*) 9-repeat allele had significantly higher increase in the levels of psychotic symptoms after acute exposure to delta-9-THC compared to subjects with other genotypes.

In single studies, the effects of interactions between cannabis use and polymorphisms in the genes encoding dopamine D2 receptor (*DRD2*) [29], FK506-binding protein 5 (*FKBP5*) [34], and brain-derived neurotrophic factor (*BDNF*) [50] were investigated. In a case-control study of FEP patients, Colizzi et al. [29] found that cannabis users with the *DRD2* rs1076560 T allele had a threefold increase in psychosis risk compared to GG homozygotes. In daily users, T allele carriers had a fivefold increase in psychosis risk compared to GG homozygotes. In healthy subjects, daily users with T allele had higher schizotypy compared to cannabis-naïve T allele carriers, cannabis users with GG genotype and cannabis-naïve GG homozygotes. Cannabis users with T allele had lower working memory performance in comparison with other groups. Ajnakina et al. [34] studied the effects of the rs1360780 polymorphism in the *FKBP5* gene in 291 first-episode psychosis patients and 218 controls. They found the association between this polymorphism and psychosis risk after co-varying for environmental factors including parental separation and cannabis use. However, no significant interactions between genetic variation in *FKBP5* gene and cannabis use on psychosis risk were found. Finally, in female schizophrenia patients, cannabis use was associated with earlier age of psychosis onset in the *BDNF* 66Met allele carriers, but not in the *BDNF* 66Val/Val homozygotes. In male patients, cannabis use was associated with earlier age of psychosis onset, regardless of the *BDNF* Val66Met genotype. The main effect of the *BDNF* Val66Met genotype on age of psychosis onset was not significant in the whole group as well as in males and females.

Interactions between genetic factors and cannabis use were tested only in one study on BD patients [101]. Authors found that the interaction between the serotonin-transporter-linked polymorphic region (5-HTTLPR) S allele and childhood sexual abuse increased odds of cannabis abuse or dependence. Cannabis abuse or dependence and the 5-HTTLPR S allele, but not childhood sexual abuse, were significantly more frequent in those patients with lifetime occurrence of psychotic symptoms.

### Gene × Stress Interactions

According to our review, 12 studies have evaluated interactions between exposure to childhood trauma and genetic factors in schizophrenia spectrum disorders [21, 22, 31, 33–36, 38, 41, 42, 45, 123]. The vast majority of studies suggest significant interactions between genetic underpinnings and exposure to childhood adversities, stressful life events or recent stressors and genetic factors, with only three studies reporting negative findings [25, 35, 45].

In three studies [21, 33, 123], interactions between a history of childhood trauma and the *BDNF* Val66Met polymorphism were addressed, showing positive findings. Aas et al. [33] found an additive effect of the *BDNF* 66Met allele and a history of childhood trauma on reduced levels of *BDNF* mRNA as well as CA2/3 and CA4 subfield volumes of dentate gyrus in the hippocampus. Similar effects of interactions between the *BDNF* Val66Met polymorphism and childhood abuse, but not childhood neglect, on positive psychotic-like experiences have been found in a non-clinical study [123]. Another non-clinical study revealed that the *BDNF* 66Val allele, especially in male twins, was associated with higher vulnerability of the effects of childhood trauma on psychotic experiences, while in the group of female twins this association was driven by the *BDNF* 66Met allele [21]. There are also studies addressing the effects of interaction between variability in the *FKBP5* gene and childhood trauma on psychosis phenotypes in clinical and non-clinical populations [22, 31, 34, 38]. In the study by Cristobal-Narvaez et al. [22], there was a significant effect of the interaction between the *FKBP5* risk haplotype (composed of three SNPs: rs3800373, rs9296158, and rs1360780) and childhood bullying on positive psychotic-like experiences, paranoia, and negative affect. The childhood bullying × *FKBP5* haplotype interaction moderated the association of social stress appraisal with psychotic-like experiences and negative affect in daily life. Specifically, this association was significantly increased in those with the risk haplotype, but not in individuals without the risk haplotype. Collip et al. [38] studied the effects of SNPs in the *FKBP5* gene (rs9296158, rs4713916, rs1043805, and rs1360780) in the general population twins, patients with psychosis, unaffected siblings of patients with psychosis and matched controls. The authors found a significant interaction between the rs9296158 and rs4713916 polymorphisms and childhood trauma on psychotic symptoms and cortisol levels in the twin sample. Similar findings were obtained for the rs4713916 polymorphism in siblings and for rs9296158 in patients. Specifically, the A allele carriers at both polymorphisms were most vulnerable to childhood trauma. In a case-control study of FEP patients mentioned above, Ajnakina et al. [34] demonstrated that the *FKBP5* rs1360780 polymorphism was associated with the risk of psychosis only after adjustment for environmental factors. Authors reported a significant effect of the interaction between the *FKBP5* rs1360780 polymorphism and parental separation on psychosis risk. In one study [31], the *FKBP5* gene polymorphisms were studied with respect to cognitive performance in patients with schizophrenia and healthy controls. Authors found significant main effects of the rs1360870 genotype and childhood trauma as well as a significant interaction between these variables affecting attention in both groups (CC homozygotes performed worse in the context of childhood trauma). Additionally, there were significant main effects of this polymorphism on global cognition in schizophrenia patients (TT homozygotes performed worse). Furthermore, McCarthy-Jones et al. [36] found in a large sample of schizophrenia spectrum patients, the interaction between variation in the forkhead box protein 2 (*FOXP2*) gene and childhood trauma in predicting a lifetime history of auditory verbal hallucinations. Emotional abuse was found to interact with the rs1456031 polymorphism in patients with CC genotype in predicting higher levels of auditory verbal hallucinations.

Another line of studies focused on the interactions between recent or daily life stressors and genetic factors [24, 37, 46, 62, 63, 65, 124]. In the majority of these studies, the effects of variation in the *COMT* gene were addressed [46, 63, 65, 124]. Van Winkel et al. [65] found that subjects with the *COMT* 158 Met/Met genotype had greater increase in overall psychotic experiences in response to daily stressors compared to those with Val/Met and Val/Val genotypes, both among patients with psychosis and healthy controls. Similarly, in the study of patients with non-affective psychosis [46], which also included the analysis of the methylenetetrahydrofolate reductase (*MTHFR*) gene polymorphisms (C677T and A1298C variants), patients with the *MTHFR* T allele, *COMT* Met/Met homozygotes showed the largest increases in psychotic experiences in response to stress. In patients, who were the *MTHFR* CC homozygotes, there was no interaction between the *COMT* Val158Met polymorphism and stress on psychotic experiences. There was also no moderating effect of the *MTHFR* A1298C polymorphism on the interaction between the *COMT* Val158Met polymorphism and stress. On the contrary, a study of female twins revealed that carriers of the *COMT* 158 Val allele displayed more feelings of paranoia in response to event stress compared with Met carriers [63]. This study also included effects of the *BDNF* Val66Met polymorphism, showing that carriers of the *BDNF* 66Met allele presented more social stress-induced paranoia than individuals with the Val/Val genotype. Similarly, Stefanis et al. [124] revealed that carriers of the *COMT* 158Val allele were more sensitive to psychosis inducing effects of stress exposure during army recruitment in comparison with the Met/Met homozygotes.

Finally, single studies focused on interacting effects of genes involved in DNA methylation, the *BDNF* gene and the neuregulin 1 (*NRG1*) gene. Pishva et al. [37], who studied the effects of DNA methylation genes in clinical and non-clinical samples, found that three SNPs in the DNA methyltransferase 3A (*DNMT3A*) gene (rs11683424, rs1465764, rs1465825) and one in the *MTHFR* (rs1801131) moderated the effect of stressful events on negative affect. The effects of the *DNMT3A* rs11683424 polymorphism were consistent in the majority of studied samples. An interesting outcome measure has been selected in the study by Gattere et al. [24], who assessed caloric intake in patients with early psychosis, ARMS individuals and healthy controls with respect to the *BDNF* Val66Met polymorphism. Authors found that perceived stress was not associated with calorie intake in healthy controls. ARMS subjects with the Met allele and low perceived stress presented with increased caloric intake, while those with high levels of perceived stress presented with decreased caloric intake. In patients with early psychosis, perceived stress was not associated with calorie intake. Perceived stress was associated with food craving in patients with psychosis. A similar association was present in ARMS subjects and healthy controls who were Val/Val homozygotes. Finally, Keri et al. [62] studied the effects of variation in the *NRG1* gene with respect to psychosocial stress in terms of conflict-related family interactions. Authors explored odd and unusual thought content during neutral and conflict-related family interactions with one of the family members: mothers, fathers, wives, husbands, and siblings in patients with schizophrenia. Patients with the *NRG1* rs6994992 TT genotype showed more unusual thoughts during conflict-related interactions than patients with CT and CC genotypes. There were no significant differences between the *NRG1* CT and CC patients. There were also no significant differences among patients with different *NRG1* genotypes during neutral interactions.

Effects of interactions between genetic factors and stressful experiences on clinical characteristics of patients with BD have been tested in six studies [101–103, 107, 108, 125]. All of these studies examined the impact of childhood trauma except for the study by Hosang et al. [102], which assessed traumatic life events. Negative results were published only by Breen et al. [107], who found no significant interactions between variation in hypothalamus-pituitary-adrenal (HPA) axis genes and childhood sexual or physical abuse on lifetime occurrence of suicide attempts. In this study, effects of the *BDNF* Val66Met polymorphism with respect to lifetime traumatic experiences and the severity of worst episodes of BD were explored. Authors revealed that the *BDNF* 66Met allele carriers with higher levels of stressful life events had a higher severity of the worst depression ever. The effects of the *BDNF* Val66Met polymorphism were also examined in the study by Miller et al. [125], who demonstrated that the *BDNF* 66Met allele carriers with a history of childhood sexual abuse had significantly higher BD severity and chronicity as well as earlier age of onset. However, these results appeared to be insignificant after controlling for potential confounders. In three studies [101, 103, 126], the impact of variation in monoaminergic genes was addressed. De Pradier et al. [101] found that the interaction between the *5-HTTLPR* S allele and childhood sexual abuse increased odds of cannabis abuse or dependence. Another study [103] revealed that the *COMT* 158Val allele was associated with higher levels of schizotypy in BD patients exposed to higher levels of childhood trauma. There were no significant main effects of the *COMT* Val158Met polymorphism on the levels of schizotypy. Finally, in one study [108], patients with the toll-like receptor 2 (*TLR2*) rs3804099 TT genotype and a history of sexual abuse had earlier age of BD onset compared to other patients. However, these results were non-significant after taking into account the effects of potential confounders.

### Gene × Season of Birth Interactions

Interactions between genetic factors and seasonality of birth were tested in four studies on patients with schizophrenia spectrum disorders [54, 71–73]. In the study by Narita et al. [73], the presence of the *HLA-DR1* allele was associated with increased incidence of winter births (February–March) in schizophrenia patients. In turn, Tochigi et al. [72] revealed no significant association between genetic variation in the *HLA-A* gene (A24 and A26 variants) and winter birth (December–March) in schizophrenia patients. Chotai et al. [71] investigating three SNPs—one in the tryptophan hydroxylase 1 (*TPH1*) gene (A218C), *5-HTTLPR* L/S polymorphism, and the dopamine D4 receptor (*DRD4*) 7-repeat allele polymorphism—demonstrated that the frequency of the *DRD4* 7-repeat allele showed one-cyclic season of birth variation in women with schizophrenia. However, no significant interactions were found for the *TPH1* A218C and *5-HTTLPR* L/S polymorphisms. In the study by Muntjewerff et al. [54], there was no significant interaction between the *MTHFR* C677T polymorphism and winter birth on schizophrenia susceptibility.

There are two studies looking into the interaction between seasonality of birth and genetic factors in BD [71, 104]. The abovementioned study by Chotai et al. [71] also included patients with BD demonstrating that allelic frequencies did not show any significant variation with respect to seasons of birth defined as four 3-month periods beginning in January. However, the analysis of one-cyclic month of birth variations showed that the *TPH1* allele A had a positive peak around the birth month December and a negative peak around June in men with BD, but not in women with BD. There were more cases of BD among men with the *TPH1* allele A born in between November and January and less cases of BD among women with the *TPH1* allele A born between February and July in comparison with healthy controls. Moreover, analysis of two cycles per year showed differences in the DRD4 gene variations both among women and men with BD. Finally, in one study the *HLA-G* 14 bp ins/del polymorphism was investigated with respect to seasonality of birth in patients with BD [104]. The authors found that the *HLA-G* ins/ins genotype was significantly less frequent in patients with BD. The prevalence of this genotype was significantly lower in patients born in the winter season.

### Gene × Infectious Factors

Interactions between genetic and infectious factors were tested in two studies on schizophrenia spectrum phenotypes [51, 66]. The study by Demontis et al. [51] revealed significant effects of the interactions between two SNPs in the glutamate ionotropic receptor NMDA type subunit 2B (*GRIN2B*) gene (rs1805539 and rs1806205) and maternal herpes simplex virus type 2 (HSV-2) seropositivity on schizophrenia risk. The latter one [66] demonstrated that variation in the MHC Class I Polypeptide-Related Sequence B (*MICB*) gene may interact with cytomegalovirus (CMV) and herpes simplex virus type 1 (HSV-1) seropositivity, influencing schizophrenia susceptibility.

In two studies on BD patients [100, 110], the presence of antibodies to HSV-1 and *Toxoplasma gondii* was analyzed. Dickerson et al. [100] found that the *COMT* 158Val/Val genotype and HSV-1 seropositivity were independent predictors of lower global cognitive performance in patients with BD. Patients with both the *COMT* 158Val/Val genotype and HSV-1 seropositivity were 85 times more likely to be in the lowest quintile of global cognitive performance. In turn, Oliveira et al. [110] revealed a trend toward significant interaction between *Toxoplasma gondii* seropositivity and the *TLR2* (rs3804099) gene polymorphism in conferring the risk of BD.

### Gene × Obstetric Complications Interactions

Interactions between genetic factors and obstetric complications were assessed in four studies on schizophrenia patients [27, 32, 56, 64]. In the study by Nicodemus et al. [64], patients with schizophrenia spectrum disorders and obstetric complications were more likely to have minor allele at the *AKT1* rs2494735 and rs1130233 polymorphisms, major allele at the BDNF rs2049046 polymorphism and minor allele at the *BDNF* rs76882600, minor allele at the dystrobrevin binding protein 1 (*DTNBP1*) gene (rs875462), and minor allele at the glutamate metabotropic receptor 3 (*GRM3*) gene (rs7808623). In turn, Ursini et al. [27] revealed that the *BDNF* Val66Met polymorphism, together with DNA methylation within this polymorphic site, might interact with obstetric complications influencing intermediate schizophrenia phenotypes, such as working memory impairment and alterations in dorsolateral prefrontal cortex activity. Haukvik et al. [56] revealed a significant effect of the interaction between the *GRM3* rs13242038 polymorphism and severe obstetric complications on hippocampal volumes in patients with schizophrenia and healthy controls. Finally, in one study [32], high birth weight was associated with schizophrenia risk in subjects homozygous for risk alleles in a four-SNP haplotype spanning the NudE Neurodevelopment Protein 1 (*NDE1*) gene and one of its constituent SNPs (rs4781678).

## Discussion

The majority of studies addressing G × E interactions in schizophrenia spectrum phenotypes and BD have focused on the effect of variation in the *COMT*, *BDNF*, and *FKBP5* genes, showing interactions with cannabis use and childhood trauma. Results of studies described in this systematic review should be discussed in frame of distinct G × E interactions models that might explain various scenarios of causality: (1) the genotype gives rise to the phenotype as the consequence of environmental exposure; however, when a risk genotype is not present, the phenotype might be expressed in case of a high-level exposure to environmental factor; (2) the genotype increases effects of environmental risk factor; however, when environmental exposure does not appear, the effects of genotype expression remain silent; (3) the environmental factor increases the effects of the high-risk genotype, but not the effects of the low-risk genotype; (4) both genetic and environmental risk factors are required to trigger the expression of the phenotype; and (5) both environmental and genetic risk factors have some effect on the phenotype; however, if they appear together the risk is higher or lower than in the situation, when they occur alone [18].

The effects of G × E interactions become even more complicated in psychiatric research, when the impact of genetic variation on personality traits, which conditions risky behaviors, is taken into account. Indeed, it might be hypothesized that some genetic factors might make individuals more prone to engage in high-risk environments [127]. This scenario might be theoretically relevant for studies investigating interactions between the *COMT* Val158Met polymorphism and cannabis use. The Val allele at codon 158 is known to increase enzymatic activity and lead to a faster breakdown of dopamine [128]. It has been hypothesized that the Val allele increases the risk of psychosis via depleting prefrontal dopamine availability that in turn increases mesolimbic dopaminergic activity in a feedback loop [129]. In turn, Δ9-tetrahydrocannabinol (THC), an active ingredient of *Cannabis sativa*, is known to enhance mesolimbic dopaminergic activity contributing to the development of psychosis [130]. Hypodopaminergic prefrontal activity, which is closely related to blunted reward processing and cognitive decline [131], has been widely observed in subjects at risk of psychosis and might explain high levels of comorbidity between cannabis abuse or dependence and schizophrenia spectrum disorders.

Although a few repeatedly tested G × E interactions can be indicated based on this systematic review, current evidence does not allow to generalize findings due to methodological heterogeneity and limitations as well as a variety of explored outcome variables. One of the main methodological problems of studies addressing G × E is sample size. Roughly speaking, a general approach states that sample sizes required to detect interactions should be at least four times higher compared to sample sizes of studies that aim to detect main effects of comparable magnitude [132, 133]. However, precise calculations that take into account genotype distribution, frequency of environmental exposure, precision of measurement, and validity of hypothesis behind a potential interaction (for a summary of various approaches see [134]), might greatly improve statistical power. Interesting simulations have been made by Uher [135], who demonstrated for instance that an interaction of moderate effect size with the genotype that is present in only 5% of the population would require 5200 participants to achieve the power of 80%. However, such requirements are a function of measurement reliability of environmental exposure. For instance, a decrease of 20% in the reliability of environment measures equates to losing about 50% of the sample. This simulation shows the importance of measurement accuracy in designing studies on G × E interactions.

Previous studies on G × E interactions have used a variety of measures for assessment of environmental exposure. The majority of them have focused on retrospective and self-report measures. This approach is particularly controversial in light of potential reporting bias driven by self-reports of childhood trauma or substance use. Previous studies with longer test-retest intervals have revealed that inconsistency rates in case of childhood trauma self-reports might reach nearly 40% [136, 137]. These studies have suggested that such factors as age, educational attainment, depressive symptoms, psychological distress, and chronic stress might impact consistency self-reports of childhood trauma. On the contrary, the Aetiology and Ethnicity of Schizophrenia and Other Psychoses (ÆSOP) study demonstrated that self-reports of childhood trauma in FEP patients remained stable over a 7-year follow-up period and were not influenced by the levels of depressive and psychotic symptoms [138]. Moreover, there are longitudinal studies that have found exposure to stressful events before the measurement of psychotic experiences or onset of psychotic disorder [139–141]. Therefore, caution should be taken on the way childhood adversities are assessed. For instance, variables that have been associated with reporting consistency should be included in statistical models analyzing G × E interactions. It has been suggested that measurement accuracy in case of childhood trauma might be increased by combining different sources of information, such as self-reports, case notes, or court records [142]. In turn, reporting accuracy of current or recent substance use might be improved by inclusion of urinary screening tests.

Another important point in G × E interactions research is that a number of individual characteristics and environmental factors are closely interrelated. For instance, it has been proposed that known schizophrenia risk factors, such as childhood trauma, urban upbringing, low intelligence quotient, migration and substance use have a common denominator—social defeat, which is defined as individual appraisal of being excluded from the society [143]. Moreover, it has been shown that childhood adversities combine with subsequent cannabis use and further increase odds of psychotic experiences—the effect described as an environment × environment interaction [144, 145]. The social defeat paradigm points to the consideration, whether environmental risk factors for schizophrenia should be captured in collective measures. Emerging evidence shows that the development of the so-called polyenviromic risk scores [146] might hold a great promise in recognizing a missing environmental contribution. This approach has been successfully implemented in the field of GWASs studies, which developed the polygenic risk score, showing that this measure is associated with schizophrenia risk and its clinical characteristics [147]. A recent study by Padmanabhan et al. [146], for the first time, demonstrated that an aggregate score of environmental exposures (winter or spring birth, cannabis abuse, advanced paternal age, obstetric and perinatal complications, physical and sexual abuse, neglect, and parental death) predicted conversion from familial high-risk state to psychosis [146].

It should be also noted that the impact of environmental exposure might be different in distinct time points of brain development, at least theoretically. Indeed, it is widely known that various neurodevelopmental processes, such as neurogenesis, neuronal migration, gliogenesis, synaptogenesis, myelination, and synaptic pruning have specific critical windows in prenatal and postnatal development [148, 149]. Seasonality of birth, advanced paternal age, and obstetric complications are considered to act as early insults and may share similar mechanisms, contributing to the pathogenesis of schizophrenia [150]. Seasonality of birth, a proxy measure of prenatal infections, might increase the risk of schizophrenia in offspring via maternal immune activation. It has been shown that elevated maternal levels of cytokines might be associated with increased risk of schizophrenia in the offspring [151, 152]. Changes in brain regions that are typical for schizophrenia patients, such as reduced cortical thickness, decreased hippocampal, prefrontal cortical and striatal volumes, and enlarged ventricles, together with reduced density of Purkinje neurons, have been also observed in the adult offspring from animal studies of maternal immune activation [153]. In addition, maternal immune activation has been found to impact dopaminergic and serotoninergic neurotransmission [153]. Obstetric complications leading to hypoxia might also indirectly act via immune-inflammatory mechanisms [150]. Another hypothesis is that hypoxia may induce expression of schizophrenia susceptibility genes. Interestingly, a recent systematic analysis of schizophrenia susceptibility genes revealed that 55% of candidate genes might be associated with ischemia-hypoxia response [154]. Further support for the association with perinatal hypoxia originates from neuroimaging studies, showing reduced gray matter volumes and increased cerebrospinal fluid space in schizophrenia patients and their siblings with a history of obstetric complications [155]. Similarly, exposure to obstetric complications has been associated with lower amygdala and hippocampal volumes in patients with BD [156]. Finally, the effect of advanced paternal age on schizophrenia risk can be explained by accumulation of de novo mutations in paternal sperm DNA or epigenetic alterations [157]. Environmental factors that act in later life, including childhood trauma and cannabis abuse might also affect brain development. A history of childhood trauma has been associated with smaller volumes of distinct brain regions, including, i.e., the corpus callosum, hippocampus, and amygdala as well as distinct reward circuits [158]. Previous studies have demonstrated that early life stress may impact psychosis risk via various biological mechanisms, such as HPA axis alterations, aberrant immune-inflammatory response, dysregulation of dopaminergic neurotransmission, and epigenetic processes [159]. Similarly, cannabis abuse might impact the development of various brain regions. The psychoactive ingredient of cannabis, THC, stimulates midbrain dopaminergic activity [130, 160]. Chronic cannabis abuse has been associated with functional and structural alterations in the hippocampus and amygdala, which are the brain regions implicated in the pathophysiology of psychosis [161]. However, it is important to note that later life environmental insults have been found to act in a dose-dependent manner and are believed to differentially impact brain development in various age groups [130, 158]. This point should be considered in analyzing results of studies on G × E interactions in schizophrenia spectrum phenotypes and BD. Given that age of exposure might be important in determining the impact of environmental exposure, a simplistic analysis of traumatic stress or substance use in the childhood or the whole lifespan, using dichotomous or continuous variables, might lead to overlooking more specific G × E interactions.

Moreover, controversy appears in the comparison of relevant G x E interactions or even main effects of environmental exposure between schizophrenia and BD as well as other psychiatric disorders or non-clinical samples. Indeed, it becomes increasingly apparent that similar G × E interactions might contribute to a broad spectrum of phenotypes. Addressing this issue should be posited as one of main directions for future studies. One direction is to implement the concept of endophenotypes, which states that there are a number of intermediate phenotypes, associated with particular disorders in the population, which are observed in non-affected individuals, exert familial co-segregation and heritability, can be observed in related disorders as well as have state-independent manifestation [162–164]. Following this broad definition, several biochemical, cognitive, behavioral, neurostructural, and neurofunctional endophenotypes have been proposed for schizophrenia and BD in order to conceptualize overlapping characteristics that fall beyond a categorical distinction [3].

Overlapping G × E interactions in various psychiatric disorders also raise a question whether G × E interactions operate through single clinical outcomes or more complex mechanisms. Recent theoretical accounts have pointed out to the need of more integrative and dynamic view and propose models that may explain the mechanisms of how the G × E interactions contribute to psychosis. On the one hand, these theories need to take into account the effects of the environment on structural and functional brain characteristics, neuroendocrine functioning, attachment styles, and patterns of affective and cognitive processing that may shape risk for later psychopathology. On the other hand, mechanisms by which genetic variation may increase susceptibility to environmental stressors should also be tested. The underlying pathophysiological pathways of these mechanisms need to be established in order to develop a priori G × E research paradigms and proper methodological designs.

For instance, Howes and Murray [165] suggested that G × E interactions contribute to dopaminergic dysregulation and lead to psychotic symptoms through a mediating role of biased information processing (i.e., cognitive biases). Indeed, with regard to cannabis use, some findings suggest that cognitive dysfunctions mediate the association between cannabis use and psychotic experiences [166]. Similarly, social adversities have been suggested to shape the risk of psychosis via biased information processing, which was confirmed in the very recent study showing a full mediation of attention to threat and external attributions biases in the relationship between traumatic life events and psychosis proneness in a non-clinical sample [167]. Several studies suggest that cognitive impairments in working memory and executive functions are linked to polymorphisms in the *COMT* gene [168, 169] and this association might be the mechanism explaining G × E interactions. For instance, a recent study has shown possible epigenetic modulation of the expression of the *COMT* Val158Met polymorphism and subsequent effects on the relationship between traumatic life events and cognition in schizophrenia [170]. However, the impact of G × E interactions on biased information processing (cognitive biases) that may be specific for psychotic or BD symptoms has not been investigated so far.

Traumatic life events may also increase the risk of psychosis since they trigger disturbances in emotional and cognitive regulative processes. In this context, an interesting approach of environmental conceptualization using the experimental sampling methodology (ESM) has been adopted in some studies discussed in this systematic review. In brief, ESM can be defined as “a research procedure that aims to provide a systematic set of self-reports obtained on random occasions about what people do and feel during waking hours of a normal week” [171]. Hence, the ESM provides an excellent tool to investigate environmental factors and its dynamic impact on well-being via cognitive-emotional processes. For instance, it has been found that a history of trauma and daily life stress impact on psychosis risk through the impaired cognitive [172] and emotional regulative processes [173] as well as a lack of resilience factors. Studies addressing G × E interactions that utilized the ESM methodology may be then interpreted rather as a dynamic phenomenon, which results in disrupted regulative processes that lead to psychotic experiences, than a simple result of direct influence of G × E on psychosis. Therefore, studies in this field may greatly benefit from combining G × E interactions and potential mediating mechanisms related to cognitive-emotional regulation in predicting psychosis or BD.

Although there is a great methodological heterogeneity in previous studies on G × E interactions in schizophrenia and BD, some findings, especially related to childhood trauma and cannabis use, have been replicated. In light of a rapid progress of GWASs in the field of BD and schizophrenia, it should be recommended and expected that future studies will focus on combining data from GWASs with environmental measures to provide more comprehensive insights into G × E interactions. This approach is currently ongoing under the European Network of National Schizophrenia Networks studying Gene-Environment Interactions (EU-GEI) Initiative [174]. In addition, future studies should also include assessment of epigenetic processes that bridge a gap between environmental exposure and genetic underpinnings.
